# Fertility Research and Practice reviewer acknowledgement 2015

**DOI:** 10.1186/s40738-016-0018-5

**Published:** 2016-03-23

**Authors:** Elizabeth Ginsburg, Danny Schust

**Affiliations:** 1 Brigham and Women’s Hospital, 75 Francis St, Boston, MA 02115 USA; 2 University of Missouri, Columbia, MO 65211 USA

## Abstract

The editors of *Fertility Research and Practice* would like to thank all of our reviewers who have contributed to the journal in Volume 1 (2015).

**Aaron Styer**

United States of America

**Alexander Quass**

United States of America

**Amanda Stephens**

United States of America

**Amber Cooper**

United States of America

**Borut Kovacic**

Slovenia

**Daniel Kaser**

United States of America

**Divya Shah**

United States of America

**Elizabeth Stewart**

United States of America

**Erma Drobnis**

United States of America

**Gordon Bates**

United States of America

**Helene Bernstein**

United States of America

**Kathy Sharpe-Timms**

United States of America

**Leslie Farland**

United States of America

**Quaker Harmon**

United States of America

**Randi Goldman**

United States of America

**Ronit Machtinger**

Israel

**Sacha Krieg**

United States of America

**Sanjeeva Reddy**

India

**Serene Srouji**

United States of America

**Shruthi Mahalingaiah**

United States of America

**Takeshi Nagamatsu**

Japan

**Tony Propst**

United States of America

**Wendy Kuohung**

United States of America

**Xinna Chen**

China

